# Prevalence of Irritable Bowel Syndrome Symptoms and Associated Risk Factors Among Medical Students

**DOI:** 10.7759/cureus.82900

**Published:** 2025-04-24

**Authors:** Ayaz Ali, Afaq Shah, Amina Amina, Zalika Aisha Ruddock-Scott, Zobia Shah, Valery Lopez Rios, Nimra Abid, Nida Gul, Kashif Khan, Shah Faisal

**Affiliations:** 1 Internal Medicine, MTI-Khyber Teaching Hospital Peshawar, Peshawar, PAK; 2 Medicine, Rehman Medical Institute, Peshawar, PAK; 3 Medicine, Bacha Khan Medical College, Mardan, PAK; 4 Medicine, Caribbean Medical University, Willemstad, CUW; 5 Medicine, Shahina Jamil Teaching Hospital, Abbottabad, PAK; 6 Medicine, Institución Universitaria Visión de las Américas, Pereira, COL; 7 Surgery, Khyber Medical University Institute of Medical Sciences, Kohat, PAK; 8 Medicine, MTI-Khyber Teaching Hospital Peshawar, Peshawar, PAK; 9 Medicine, Khyber Medical College, Peshawar, PAK

**Keywords:** dietary habits, irritable bowel syndrome (ibs), medical students, prevalence, risk factors

## Abstract

Introduction

Irritable bowel syndrome (IBS) is a common functional gastrointestinal disorder characterized by recurrent abdominal pain and altered bowel habits. Medical students, due to academic stress, irregular dietary patterns, and disrupted sleep, may be at a higher risk of developing IBS. This study aims to determine the prevalence of IBS symptoms among medical students, identify associated risk factors, and assess its impact on daily activities and academic performance.

Methodology

A cross-sectional study was conducted among medical students in Peshawar, Pakistan. Data were collected using a validated questionnaire based on the Rome IV criteria for IBS. Information on demographic characteristics, stress levels, dietary habits, sleep patterns, and IBS symptoms was obtained. The sample size consisted of 480 participants selected through a stratified random sampling technique. Data were analyzed using SPSS version 20, and the chi-square test was applied to determine associations between IBS symptoms and various risk factors.

Results

The prevalence of IBS symptoms among medical students was found to be 60%. A statistically significant association was observed between IBS and gender, with female students being more affected (p < 0.05). Higher academic years had an increased prevalence of IBS symptoms compared to junior students. Additionally, students residing in hostels were more likely to report IBS symptoms than those living at home. Stress, irregular dietary habits, and poor sleep quality were identified as key contributing factors. IBS symptoms significantly impacted daily activities and academic performance in affected students.

Conclusion

This study highlights a high prevalence of IBS symptoms among medical students, with stress, dietary habits, and sleep disturbances playing a crucial role. Female students, those in senior academic years, and hostel residents exhibited a higher burden of symptoms. Early interventions focusing on stress management, dietary modifications, and lifestyle improvements are essential to mitigate IBS-related health concerns in this population.

## Introduction

In the absence of an organic intestinal lesion, irritable bowel syndrome (IBS) is a common functional gastrointestinal illness marked by frequent changes in bowel habits, abdominal pain or discomfort, and/or bloating [1] In the United States alone, between 2.4 and 3.5 million doctor visits are attributed to IBS each year, making it the most common illness identified by gastroenterologists. It is prevalent across all socioeconomic classes and societies [2].

IBS affects between 9% and 23% of people worldwide. However, depending on the diagnostic instrument employed, it differs from one nation to another. IBS affects 10% to 15% of people in North America and 10% to 20% of people in Western countries, according to Rome III criteria [3,4]. However, two distinct studies were carried out in Makkah and Aljouf, Saudi Arabia. Using the Rome III criteria, 26.7% of the individuals in Makkah received an IBS diagnosis. Using Manning criteria, the prevalence of IBS in Aljouf was 8.9%, whereas using Rome II criteria, it was 9.2% [5].

There are several symptom-based diagnostic criteria that can be used to identify functional gastrointestinal disorders (FGIDs), most of which entail persistent or recurring symptoms. Actually, no pathophysiological mechanism that causes these illnesses to accumulate has been identified as of yet. However, a number of studies have proposed certain contributing elements that enable these illnesses, with the most prevalent ones being intestinal microbes, low-grade inflammation, altered brain-gut function, visceral hypersensitivity, aberrant gastrointestinal motility, and psychosocial instability [6]. The incidence of IBS is partly a result of cultural food customs in different parts of the world. For instance, it is advised that our daily diet contains at least 20-30 g of dietary fiber. However, prior research has shown that the daily dietary fiber intake of the Western diet is substantially lower than that of the Indian subcontinent. Because of this, Western adults have a far higher prevalence of IBS than Indian adults [7].

Additionally, there is a correlation with psychological-psychiatric disorders, as IBS patients have much greater levels of anxiety and despair than healthy controls. According to a meta-analysis, the prevalence of anxiety symptoms in IBS patients is around 39.1%, whereas the prevalence of depressive symptoms is approximately 28.8% [8]. Stress has been linked to IBS as a trigger and an aggravating factor [9]. Junior physicians and medical students are among the groups of people who are subjected to high levels of stress because of their diverse obligations and demanding workloads. The frequency of IBS in junior physicians and medical students varies from 9.3% to 35.5% [10].

Information from Pakistan is scarce. Using the Rome II criteria, a population-based study from Abbottabad, Pakistan, reported that 13% of people had IBS. Age and sex have an impact on IBS, which is a multifactorial illness. Early adulthood is when symptoms first arise, but as people age, they lessen. IBS primarily affects those under the age of 25 [11]. According to the literature, IBS is more common in women than in men, though it is unclear why. However, according to an IBS study conducted in India, 6.9% of women and 7.9% of men have IBS [12,13].

IBS is a prevalent FGID that significantly impacts individuals' quality of life, particularly among young adults, including medical students who are often exposed to high levels of academic stress, irregular dietary habits, and disrupted sleep patterns. Given the demanding nature of medical education, students may be more susceptible to IBS symptoms, which can further interfere with their academic performance and daily functioning. This study aims to determine the prevalence of IBS symptoms among medical students while also exploring associated contributing factors such as stress, dietary habits, and sleep disturbances. Additionally, it seeks to evaluate how IBS affects students' academic and personal lives and to identify potential variations in prevalence across different academic years and genders. Understanding these aspects can help design targeted interventions to improve students' gastrointestinal and overall well-being.

## Materials and methods

This study was conducted as a cross-sectional analysis to determine the prevalence of IBS symptoms and associated risk factors among medical students in Peshawar. The cross-sectional design was chosen to capture a snapshot of IBS prevalence and its relationship with various risk factors at a single point in time. The research was carried out across multiple medical colleges in Peshawar, including Khyber Medical College (KMC), Rehman Medical Institute (RMI), Northwest School of Medicine, Khyber Girls Medical College (KGMC), and Peshawar Medical College (PMC). These institutions were selected to ensure diverse representation and improve the generalizability of findings across different student populations. The study population comprised MBBS students from the first year to the final year, as this group is commonly exposed to high academic stress, irregular dietary habits, and varying sleep patterns - factors that may contribute to the development of IBS. A total of 480 students participated in the study, a sample size deemed sufficient to provide a reliable estimate of IBS prevalence and associated risk factors. A random sampling technique was used to select participants from each medical college to ensure an unbiased and representative selection process across different academic years. Data were collected through a structured questionnaire consisting of sections on demographic details (such as age, gender, and year of study), the presence of IBS symptoms, dietary habits, stress levels, sleep patterns, and the impact of IBS on academic performance. The diagnosis of IBS was established using the Rome IV criteria, widely accepted diagnostic tools for identifying IBS, and students meeting the criteria were classified as having IBS.

Data analysis

The collected data were entered and analyzed using SPSS software (IBM Corp., Armonk, NY). Frequencies and percentages were calculated to determine the prevalence of IBS symptoms. The chi-square test was used to assess the association between IBS symptoms and various risk factors such as stress, dietary habits, and sleep patterns. Statistical significance was set at p < 0.05 (the chi-square test was used to find the p-value), ensuring that the findings were meaningful and not due to random chance.

## Results

Among the 192 (40%) male students, 48 (25%) reported IBS symptoms, while 144 (75%) did not. In contrast, out of the 288 (60%) female students, 250 (86%) had IBS symptoms, whereas only 38 (14%) were symptom-free. A p-value of 0.001 indicates a statistically significant association between gender and IBS prevalence, with females experiencing IBS symptoms at a much higher rate compared to males.

When analyzing IBS prevalence across different academic years, a rising trend was observed as students progressed through medical school. Among first-year students (90 [18%]), 30 (33%) had IBS, while in second-year students (102; [21%]), 34 (34%) reported IBS symptoms. The prevalence increased in third-year students (58 [12%]), with 40 (68%) affected, and in fourth-year students (86 [17%]), where 60 (70%) had IBS. Final-year students (144 [30%]) exhibited the highest prevalence, with 122 (84%) experiencing IBS symptoms. A p-value of 0.001 suggested a significant correlation between academic year and IBS prevalence, highlighting a progressive increase in IBS cases among higher-year students.

Regarding residency status, IBS symptoms were more prevalent among students living in college dormitories. Out of 336 (70%) hostel residents, 240 (71%) reported IBS symptoms, whereas 96 (29%) were symptom-free. Among 144 (30%) day scholars, 86 (59%) had IBS symptoms, while 58 (41%) did not. A p-value of 0.001 indicates a statistically significant relationship between residency and IBS prevalence, suggesting that hostel residents are more affected than day scholars. Overall, the statistically significant p-values across all variables reinforce the strong association of IBS symptoms with gender, academic year, and residency status (Table 1).

**Table 1:** Association of IBS symptoms with gender, academic year, and residency status among medical student IBS, irritable bowel syndrome; n, frequency; %, percentage

Variables	Category	Frequency	percentage	IBS symptoms present, n (%)	IBS symptoms absent, n (%)	p-Value (significant when less than 0.05)	Chi-square value
Gender	Male	192	40%	48(25%)	144 (75%)	0.001	163.33
	Female	288	60%	250(86%)	38 (14%)		
Year of MBBS	1^st^ year	90	18%	30 (33%)	60 (67%)	0.001	46.66
	2^nd^ year	102	21%	34 (34%)	66 (66%)		
	3^rd^ year	58	12%	40 (68%)	18 (32%)		
	4^th^ year	86	17%	60 (70%)	26 (30%)		
	5^th^ year	144	30%	122 (84%)	22 (16%)		
Residency	Hostel residents (resident in college dormitories)	336	70%	240 (71%)	48 (29%)	0.001	60.95
	Day scholars (living with their families)	144	30%	86 (59%)	106 (41%)		

Among the participants, 60% (n = 288) reported experiencing recurrent abdominal pain at least once a week in the past three months, while 40% (n = 192) did not experience such symptoms. This indicates a significant proportion of students suffering from abdominal discomfort, a key diagnostic feature of IBS.

Regarding bloating, which is another common symptom, 53.75% (n = 258) of students reported experiencing bloating frequently, 31.25% (n = 150) experienced it occasionally, and only 14.5% (n = 70) reported never feeling bloated. This suggests that bloating is a prevalent issue among medical students, which may be linked to dietary habits, stress, or lifestyle factors.

When asked about changes in bowel habits (diarrhea or constipation) along with abdominal pain, 64.5% (n = 310) reported experiencing these symptoms, whereas 35.5% (n = 170) did not. The high prevalence of altered bowel habits among students aligns with the diagnostic criteria for IBS and indicates a substantial burden of gastrointestinal discomfort in this population.

Overall, these findings suggest a high prevalence of IBS-related symptoms among medical students, which may warrant further investigation into contributing factors such as stress, diet, and lifestyle choices (Table 2).

**Table 2:** Frequency distribution of IBS symptoms among medical students

Variables	Category	Frequency	Percentage %
In the past 3 months, have you experienced recurrent abdominal pain (at least once a week)?	Yes	288	60%
	No	192	40%
How often do you feel bloated?	Never	70	14.5%
	Occasionally	150	31.25%
	Frequently	258	53.75%
Do you experience changes in bowel habits (diarrhea or constipation) along with abdominal pain?	Yes	310	64.5%
	No	170	35.5%

A significant relationship was observed between academic stress and IBS symptoms (p = 0.001). Among students who reported experiencing academic stress "always" (n = 116) (40%) and "often" (n = 96) (33%), the prevalence of IBS symptoms was notably high. In contrast, those who reported "never" experiencing academic stress (n = 6) (2%) had the lowest prevalence of IBS, with the majority (n = 144) (75%) being symptom-free. This strong association suggests that higher stress levels may be a key contributing factor to IBS symptoms.

Similarly, sleep duration showed a significant correlation with IBS symptoms (p = 0.001). Students sleeping less than four hours per night had the highest prevalence of IBS (n = 140) (48.6%), while those who slept more than eight hours had the lowest (n = 52) (18%). A large proportion of students with sufficient sleep (n = 144) (76%) remained IBS-free, suggesting that adequate sleep may serve as a protective factor against IBS.

Physical activity demonstrated a strong inverse relationship with IBS prevalence (p = 0.001). Among students who never engaged in physical activity, 63.8% (n = 184) had IBS symptoms, while only 5% (n = 10) remained symptom-free. In contrast, students who exercised three or more times per week had the lowest IBS prevalence (n = 28) (10%), with 76.3% (n = 144) being free of IBS symptoms. This suggests that regular exercise may help reduce IBS risk, potentially due to its role in stress relief and improved gut motility.

**Figure 1:**
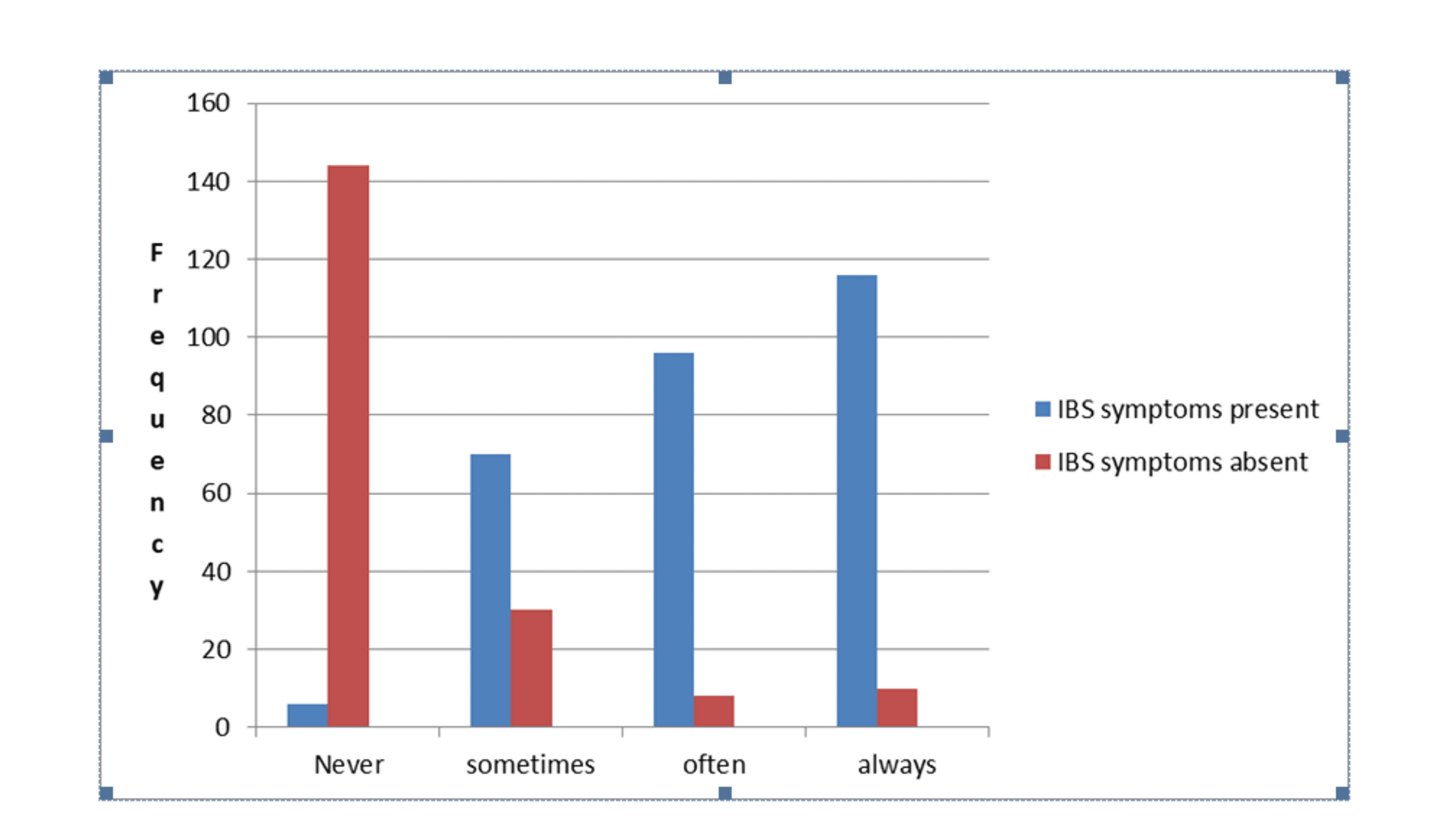
Association of IBS symptoms with risk factors among medical students IBS, irritable bowel syndrome

Dietary habits, particularly fast food consumption, were also significantly associated with IBS symptoms (p = 0.001). Students who consumed fast food three or more times per week had the highest prevalence of IBS (n = 192) (66.5%), whereas those who never consumed fast food had the lowest (n = 8) (3%). Among students who did not consume fast food, a significant majority (n = 164) (85.4%) were free from IBS symptoms. These findings suggest that an unhealthy diet may exacerbate IBS symptoms, reinforcing the importance of balanced dietary habits in maintaining gut health (Table 3, Figure 1). Overall, the statistical significance (p-value = 0.001) for all associations confirms that academic stress, poor sleep, lack of physical activity, and frequent fast food consumption are strongly linked to IBS symptoms in medical students. These findings highlight the importance of stress management, maintaining a healthy sleep schedule, engaging in regular physical activity, and following a balanced diet to potentially reduce IBS prevalence in this population.

**Table 3:** Association of IBS symptoms with risk factors among medical students IBS, irritable bowel syndrome; n, frequency; %, percentage

Risk factors	Category	IBS symptoms present, n (%)	IBS symptoms absent, n (%)	p-Value (significant when less than 0.05)	Chi-square value
Academic stress	Never	6 (2%)	144 (75%)	0.001	240.000
	Sometimes	70 (24%)	30 (15%)		
	Often	96 (33%)	8 (4%)		
	Always	116 (40%)	10 (5%)		
Sleep duration	Less than 4 hours	140 (48.6%)	4 (2%)	0.001	106.677
	4 to 6 hours	96 (33.4%)	44 (22)		
	More than 8 hours	52 (18%	144 (76%)		
Physical activity	Never	184 (63.8%)	10 (5%)	0.001	180.00
	1 to 2 times per week	76 (26.3%)	38 (19.7%)		
	3 or more times per week	28 (10%)	144 (76.3%)		
Fast food consumption	Never	8 (3%)	164 (85.4%)	0.001	163.66
	1 to 2 times per week	88 (30.5%)	20 (10.6%)		
	3 or more times per week	192 (66.5%)	8 (4%)		

The analysis revealed a significant impact of IBS symptoms on the academic performance and study concentration of medical students. Among students experiencing IBS symptoms, 70% (n = 200) reported difficulty concentrating on their studies, while only 30% (n = 88) stated that their concentration remained unaffected. In contrast, among those without IBS symptoms, only 22% (n = 48) reported concentration difficulties, whereas the majority, 78% (n = 144), did not face such issues. This difference was statistically significant (p = 0.001), suggesting a strong association between IBS symptoms and reduced study concentration.

**Figure 2:**
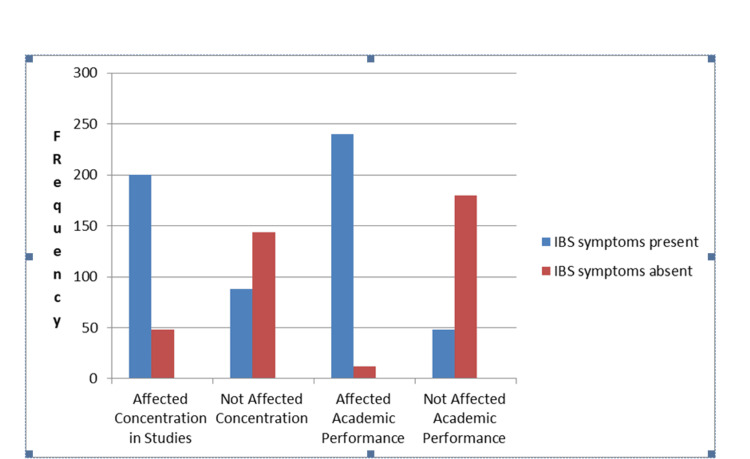
Impact of IBS symptoms on academic performance and daily study routine among medical students IBS, irritable bowel syndrome

Furthermore, IBS symptoms also had a notable effect on academic performance. Among IBS-affected students, 83% (n = 240) reported a negative impact on their academic performance, while only 17% (n = 48) stated that their performance remained unaffected. Conversely, among students without IBS symptoms, only 6% (n = 12) experienced academic difficulties, whereas the majority, 94% (n = 180), reported no adverse impact on their performance. This association was also statistically significant (p = 0.001) (Table 4, Figure 2). These findings highlight that IBS symptoms significantly impair both concentration and academic performance, emphasizing the need for better awareness, management strategies, and support systems for students dealing with IBS-related challenges.

**Table 4:** Impact of IBS symptoms on academic performance and daily study routine among medical students IBS, irritable bowel syndrome; n, frequency; %, percentage

IBS symptoms present	Affected concentration in studies, n (%)	Did not affect concentration, n (%)	Affected academic performance, n (%)	Did not affect academic performance, n (%)	p-Value (significant when less than 0.05)	Chi-square value
Yes	200 (70%)	88 (30%)	240 (83%)	48 (17%)	0.001	167.00
No	48 (22%)	144 (78%)	12 (6%)	180 (94%)	0.001	167.00

## Discussion

The findings of this study indicate a significant prevalence of IBS symptoms among medical students, highlighting the impact of various lifestyle and psychological factors. Female students reported a higher prevalence of IBS symptoms compared to their male counterparts, suggesting a possible role of hormonal influences and gender-specific stress responses. Additionally, senior-year students exhibited a greater burden of IBS, likely due to increased academic pressure and workload as they progress through medical school. Hostel residents were more affected than students living at home, which may be attributed to irregular meal patterns, higher stress levels, and differences in dietary quality. A strong association was observed between IBS symptoms and stress, reinforcing the role of psychological distress in exacerbating gastrointestinal disturbances. Furthermore, students with irregular dietary habits, including frequent consumption of fast food and caffeine, had a higher likelihood of experiencing IBS symptoms. Poor sleep quality was another key contributing factor, with students who reported inadequate sleep experiencing more frequent and severe IBS-related complaints. The impact of IBS on daily activities and academic performance was substantial, as affected students frequently reported difficulty concentrating, absenteeism, and reduced productivity. These findings underscore the need for targeted interventions to address stress management, promote healthy dietary habits, and improve sleep hygiene among medical students to mitigate IBS-related health concerns.

A study conducted in Bangladesh highlighted that IBS not only leads to physical discomfort but also contributes to psychological distress, ultimately reducing the overall quality of life [14]. Similarly, our findings reinforce this observation, as a significant proportion of medical students with IBS symptoms reported difficulties in concentration (70%) and a negative impact on academic performance (83%). These results suggest that the burden of IBS extends beyond gastrointestinal symptoms, affecting mental well-being and daily functioning. The strong association between IBS and stress in our study further supports this, emphasizing the need for stress management strategies to improve both physical and psychological health in affected individuals.

In comparison with previous studies, where the reported prevalence of IBS among medical students was 31.7%, our study found a higher prevalence of IBS symptoms (60%). This suggests a substantial burden of IBS symptoms in our study population, which may be attributed to differences in diagnostic criteria, lifestyle habits, or environmental stressors among students.

Consistent with prior research, our study also found that IBS symptoms were more prevalent among females. This aligns with existing literature that suggests hormonal and psychosocial factors may contribute to the increased susceptibility of females to IBS. Additionally, students with a positive family history of IBS were more likely to exhibit symptoms, reinforcing the role of genetic predisposition in the development of IBS [15].

One key finding in both studies is the protective effect of regular physical activity against IBS symptoms. In previous research, IBS was significantly less prevalent among students who engaged in regular exercise. Similarly, in our study, students who exercised three or more times per week had the lowest IBS prevalence (10%), whereas those who were physically inactive had the highest prevalence (63.8%). This highlights the potential benefits of physical activity in managing IBS, possibly due to its role in stress reduction and improved gut motility.

While the previous study did not report on factors such as academic stress, sleep deprivation, or dietary habits, our findings suggest that these are significant contributors to IBS prevalence. The strong associations found in our study between IBS and high academic stress, inadequate sleep, and frequent fast food consumption further emphasize the need for lifestyle modifications as a preventive strategy.

Overall, while both studies highlight the high burden of IBS symptoms among medical students, our research provides additional insight into modifiable risk factors, reinforcing the importance of stress management, adequate sleep, regular exercise, and a balanced diet in reducing IBS prevalence.

Our study aligns with previous research regarding the role of dietary factors in IBS development. An Indian study reported that the consumption of fatty foods significantly increased the risk of IBS, which is consistent with our findings [16]. In our study, students who frequently consumed fried and high-fat foods had a higher prevalence of IBS symptoms, reinforcing the association between dietary fat intake and gastrointestinal discomfort. This may be due to the impact of fatty foods on gut motility, visceral hypersensitivity, and microbiota composition, all of which are known to influence IBS symptoms.

Additionally, other studies have highlighted a strong link between spicy and salty food consumption and IBS development [17]. Our findings support this, as students who regularly consumed spicy and processed foods exhibited a higher prevalence of IBS symptoms compared to those with a more balanced diet. The irritation caused by spicy foods to the gastrointestinal mucosa, along with the possible role of high salt intake in gut inflammation and altered gut microbiota, may explain these associations.

Overall, our results reinforce the growing body of evidence that diet plays a crucial role in IBS development, with high-fat, spicy, and processed foods acting as potential triggers. These findings highlight the need for dietary modifications as part of IBS management strategies, particularly among medical students who may have irregular eating patterns due to academic stress.

Our study also supports the role of genetic factors in the development of IBS, aligning with findings from the Swedish case-cohort study, which reported that IBS clusters within families and that the familial risk increases among first-degree, second-degree, and third-degree relatives [18]. In our study, a higher prevalence of IBS symptoms was observed among students with a positive family history of IBS, reinforcing the notion that genetic predisposition plays a significant role in IBS susceptibility.

The familial aggregation observed in both studies suggests that genetic factors, shared environmental exposures, or a combination of both may contribute to the development of IBS. While the Swedish study quantified the increased odds of IBS among relatives, our findings provide further evidence of this trend in a medical student population, emphasizing the need for targeted awareness and preventive strategies among individuals with a family history of IBS.

Similar to the findings from Gondar University, our study also highlights the high prevalence of IBS among medical students, likely due to the academic stress associated with medical education [19]. We found that IBS symptoms were significantly associated with stress, sleep disturbances, and irregular meal patterns, indicating that the demanding nature of medical studies plays a crucial role in the development and exacerbation of IBS.

Furthermore, in our study, students with IBS reported reduced study hours, frequent sleep disturbances, and meal skipping, which mirrors the impact observed in the Gondar University study. These findings emphasize the need for stress management programs, dietary awareness, and lifestyle modifications to mitigate the burden of IBS among medical students.

A study has found a higher prevalence of IBS among students residing in dormitories compared to those living with their families [20], which aligns with our findings. In our study, IBS symptoms were reported by 71% of hostel residents (240 out of 336), whereas only 59% of day scholars (86 out of 144) experienced similar symptoms. The statistically significant p-value of 0.001 further reinforces this association, suggesting that students living in dormitories are more susceptible to IBS. This may be attributed to factors such as increased stress, irregular eating habits, and disrupted sleep patterns, which are more common among hostel residents.

Limitations

This study has certain limitations that should be acknowledged. Firstly, it was conducted in a single city, Peshawar, which may limit the generalizability of the findings to medical students in other regions or countries with different educational systems, lifestyles, and environmental factors. Secondly, the cross-sectional design of the study captures data at only one point in time, which restricts the ability to establish causal relationships between IBS symptoms and associated risk factors such as stress, dietary habits, and sleep patterns. Therefore, longitudinal or multi-center studies are recommended in the future to confirm these associations and provide a more comprehensive understanding of IBS among medical students.

## Conclusions

This study highlights a significant prevalence of IBS symptoms among medical students in Peshawar, with notable associations with gender, academic year, and residency status. Female students, those in higher academic years, and hostel residents reported a significantly higher burden of IBS symptoms. The findings suggest that increasing academic stress, irregular dietary habits, and disrupted sleep patterns contribute to the rising prevalence of IBS as students progressed through medical school.

Furthermore, a considerable proportion of students reported recurrent abdominal pain, bloating, and altered bowel habits, reinforcing the need for increased awareness and targeted interventions to address IBS-related concerns. Given the substantial impact of IBS on daily activities and academic performance, early identification, stress management strategies, and lifestyle modifications should be integrated into student health programs. Future studies with larger sample sizes and longitudinal designs could further elucidate the causal relationships between stress, dietary habits, and IBS symptoms in this population.

## Appendices

Questionnaire: Prevalence of IBS Symptoms Among Medical Students

Section 1: Demographic Data

Gender: ☐ Male ☐ Female

Year of MBBS: ☐ 1st ☐ 2nd ☐ 3rd ☐ 4th ☐ Final

Residence: ☐ Hostelite ☐ Day Scholar

Section 2: IBS Symptoms

In the past 3 months, have you experienced recurrent abdominal pain (at least once a week)?

☐ Yes ☐ No

Do you experience changes in bowel habits (diarrhea or constipation) along with abdominal pain?

☐ Yes ☐ No

How often do you feel bloated?

☐ Never ☐ Occasionally ☐ Frequently

Do your bowel habits interfere with your daily activities?

☐ Yes ☐ No

Have you ever been diagnosed with IBS by a doctor?

☐ Yes ☐ No

Section 3: Contributing Factors

How often do you consume fast food?

☐ Never ☐ 1-2 times/week ☐ 3+ times/week

How many glasses of water do you drink per day?

☐ < 3 ☐ 3-5 ☐ 6-8 ☐ >8

Do you experience academic stress?

☐ Never ☐ Sometimes ☐ Often ☐ Always

How many hours of sleep do you get per night?

☐ <4 ☐ 4-6 ☐ 6-8 ☐ >8

How often do you engage in physical activity (exercise/sports)?

☐ Never ☐ 1-2 times/week ☐ 3+ times/week

Do you have a family history of IBS or similar gut-related issues?

☐ Yes ☐ No ☐ Don’t know

Section 4: Impact on Academic Life

Have you missed classes due to abdominal discomfort?

☐ Yes ☐ No

Has IBS-like discomfort affected your concentration in studies?

☐ Yes ☐ No

Has IBS affected your academic performance?

☐ Yes ☐ No
